# *Dirofilaria repens*: emergence of autochthonous human infections in the Czech Republic (case reports)

**DOI:** 10.1186/s12879-016-1505-3

**Published:** 2016-04-19

**Authors:** Jana Matějů, Marta Chanová, David Modrý, Barbora Mitková, Kristýna Hrazdilová, Víta Žampachová, Libuše Kolářová

**Affiliations:** Institute of Immunology and Microbiology, First Faculty of Medicine, Charles University in Prague and General University Hospital in Prague, Prague, Czech Republic; National Reference Laboratory for Tissue Helminthoses, General University Hospital in Prague, Prague, Czech Republic; Department of Pathology and Parasitology, Faculty of Veterinary Medicine, University of Veterinary and Pharmaceutical Sciences, Brno, Czech Republic; Central European Institute for Technology, University of Veterinary and Pharmaceutical Sciences, Brno, Czech Republic; Biology Centre, Institute of Parasitology, Czech Academy of Sciences, České Budějovice, Czech Republic; Department of Virology, Veterinary Research Institute, Brno, Czech Republic; First Department of Pathological Anatomy, Faculty of Medicine, Masaryk University – St. Anne’s University Hospital Brno, Brno, Czech Republic

**Keywords:** *Dirofilaria repens*, Human dirofilariasis, Emerging disease, Autochthonous diseases in Czech Republic

## Abstract

**Background:**

Human dirofilariasis is a zoonotic infection that continues to spread to previously unaffected areas of Europe. In the South Moravian Region of the Czech Republic (CR), imported as well as autochthonous canine infections were recorded in the last decade, and parasite DNA was detected in mosquitoes of *Aedes vexans*. In the present paper, human *Dirofilaria* infections are reported from the country for the first time.

**Case presentation:**

The samples from five patients with suspected tissue helminthiases were investigated. In particular cases, nematodes were isolated from various tissues including skin of lower leg, soft tissues of finger, subcutaneous tissue of hypogastrium, lymph node and peritoneum. The diagnosis was based on light microscopic morphology and/or DNA analysis of the worms. In addition, ELISA examination of patients’ sera for anti-filaria IgG antibodies was performed.

**Conclusions:**

In the CR, five cases of human dirofilariasis caused by *Dirofilaria repens* were recorded during 2010–2014 (species determination for three of them was confirmed besides morphological also by DNA analysis). At least, three of the cases were of autochthonous origin (the patients are Czech citizens residing in South Moravian Region who have never travelled abroad). The findings confirm the natural setting of *D. repens* in South Moravian Region of the CR. Dirofilariasis should be therefore considered as endemic in this area where it may represent a significant risk factor for public health.

## Background

Dirofilariasis is a term used for the group of vector-borne parasitoses caused by worms of the genus *Dirofilaria* (Nematoda, Onchocercidae). Approximately 30 species divided in two subgenera (*Dirofilaria* and *Nochtiella*) are recognized. Different host specificity, life strategies and clinical manifestations among *Dirofilaria* spp. are reported. In Europe, two *Dirofilaria* species, *D.* (*D.*) *immitis* and *D.* (*N.*) *repens*, occur which both cause animal as well as human dirofilariasis [1]. The life cycle of *Dirofilaria* spp. involves mosquitoes and carnivores (mainly dogs and foxes, but also cats, ferrets, raccoons and bears) as intermediate and definitive hosts, respectively. The dogs represent the main natural reservoir of infection [2]. Humans are considered to be abnormal hosts, unsuitable for completion of the parasite’s life cycle. Although inoculated larvae fail to develop a productive infection, their migration from the infection site (skin) and persistence in various tissues/organs may have significant clinical consequences [2].

The symptoms of human dirofilariasis depend on the sites where the larvae have located. In many cases, the infection progresses inconspicuously with nonspecific clinical symptoms. Immune-mediated formation of nodules surrounding the larvae is the most frequent pathologic finding associated with the infection. The former strict relation between particular *Dirofilaria* species and its tissue-specific residence has recently been reconsidered, since many of the cases have rather atypical locations [2]. Skin, ocular and pulmonary dirofilariases are reported the most frequently with subcutaneous dirofilariasis being by far the major clinical presentation among human cases in Europe. It is characterized by formation of gradually growing and sometimes migrating erythematous nodules and it is mostly caused by *D. repens* [2]. These parasites are also responsible for most of the ocular infections that include nodule formation in the orbital zone and eyelids, as well as the presence of intact migrating worm in subconjunctival and intravitreous tissues [3]. Pulmonary dirofilariasis is typically associated with *D. immitis.* It is responsible for the formation of pulmonary nodules surrounding the larvae entrapped in the arterial lumen [2] and for the disruption of the arterial wall due to worm penetration with subsequent development of focal necrosis [4].

Human dirofilariasis is reported all over the world. In Europe, the highest incidence of human cases occurs in traditional endemic areas of southern countries (Italy, France, Greece) and Ukraine [5]. Recently, the disease has spread over formerly unaffected areas of Europe. Emergence of autochthonous human dirofilariasis caused by *D. repens* was recorded in Hungary (2000) [6], Austria (2006) [7], Slovakia (2008) [8], Poland (2010) [9] and Germany (2014) [10], i.e. countries neighbouring the Czech Republic (CR), as well as those that are distant, such as Serbia (2009) [11]. These findings in particular areas were usually preceded by cases of imported human dirofilariasis as well as imported and/or autochthonous canine dirofilariasis, or dirofilariasis of wild carnivores.

In the CR, the first record of dirofilariasis, particularly imported dog infection with *D. immitis*, was reported in 2003 [12]. *Dirofilaria repens* occurrence, including the first autochthonous canine infection [13] and the detection of *D. repens* DNA in the vector mosquito of *Aedes vexans* [14], was reported some years later. Currently, *D. repens* is well established in dog populations in the extreme southeast of the territory of the country [13, 15].

In the present paper, recent cases of human dirofilariasis caused by *D. repens* in the CR are reported; at least three of them evidenced as being autochthonous. The following diagnostic criteria were used: i) detection and subsequent morphology and/or DNA analysis of the worms; and, ii) detection of anti-filaria IgG antibodies in patients’ sera. Our findings add to the data on the current distribution of dirofilariasis in Central Europe.

## Methods

The samples from five patients aged 17–61 years with suspected tissue helminthiases were investigated**.** Tissue biopsies or extracted worms were obtained in local Czech hospitals. Sera of patients for specific antibodies detection were taken and supplied by attending physicians at various post-examination and postoperative intervals. The travel history and other anamnestic data were collected using a standardized questionnaire for tissue helminthiases diagnostics; clinical data and results of physical investigations were supplied by physicians; none of used data could lead to patient identification. For an overview of cases, material obtained and diagnostic method used see Table 1.

**Table 1:** Overview of patients’ travel history, examined material and diagnostic method used for particular cases

Case No	Travel history	Infection localisation	Parasite material examined	Dg method performed
1	No	Subcutaneous nodule (hypogastrium)	Paraffin embedded tissue block	M, D, S
2	No	Peritoneum	Single isolated formalin fixed worm	M, D, S
3	Yes	Skin lesion (left ankle)	Two isolated formalin fixed worms	M, S
4	No	Subcutaneous nodule (left middle finger)	Single isolated ethanol fixed worm	M, D, S
5	Yes	Lymph node	Paraffin embedded tissue block	M, S

*M* microscopy, *D* DNA analysis, *S* serological examination

Biopsies were routinely processed for histological examination (paraffin embedding, haematoxylin-eosin and periodic-acid Schiff staining) and extracted worms were observed *in toto*, both using light microscope Olympus BX 40 equipped with Olympus C-5050 ZOOM camera and M.I.S QuickPHOTO Pro software for microphotographs.

Serological examination was performed by ELISA in-house method using *Acanthocheilonema* (syn. *Dipetalonema*) *vitae* somatic antigen for detection of specific anti-filaria IgG antibodies. Confirmatory testing was performed using commercial *Acanthocheilonema vitae* ELISA kit (9400, Bordier Affinity Products SA). For exclusion of non-specific positive reactions, samples were simultaneously examined by ELISA for IgG reactivity with the scale of other parasitic antigens (*Toxocara canis, Trichinella spiralis, Echinococcus granulosus, Taenia saginata, Fasciola hepatica*) routinely used for human diagnostics.

The worm DNA was isolated from 70 % ethanol fixed samples using Genomic DNA Mini Kit (GB300, Geneaid Biotech, Taiwan); DNA from formalin-fixed and paraffin-embedded samples was isolated using High Pure PCR Template Preparation Kit (11796828001, Roche Diagnostics, Germany) according to the manufacturers’ instructions. Amplification protocol for 247 bp and 153 bp long fragments of 5S rRNA gene was adopted from Rishniw 2006 [16]. PCR products were purified using Gel/PCR DNA Fragment Extraction Kit (DF300, Geneaid Biotech, Taiwan), cloned into pGEM®-T Easy Vector System (A1360 Promega Corp., Madison, WI) according to the manufacturer’s instructions. Plasmid DNA was purified by GenElute™ Plasmid Miniprep Kit (PLN350 Sigma-Aldrich, St. Louis, MO). Cloned DNA was sequenced by Macrogen capillary sequencing services (Macrogen Europe, The Netherlands) using primer specific to SP6 promoter vector sequences. Simultaneously, PCRs amplifying *cox*1 gene fragments using the combination of single reverse primer (COIintR) with universal (COIintF) or species-specific primer (Drcox1F) were performed [17, 18]. PCR products were purified and both strands directly sequenced (Macrogen) using amplification primers.

All the procedures in the study involving human material and data were performed in accordance with WMA Declaration of Helsinki - Ethical Principles for Medical Research Involving Human Subjects; the study was approved by the Ethics committee of the General University Hospital in Prague, Czech Republic (FWA 1490000302, IRB 00002705, IORG0002175 according to the Office for Human Research Protections, U.S. Department of Health and Human Services).

## Case presentation

### Case 1 (August 2014)

A patient of Czech origin without any travel history abroad presented with a palpable mass in the right hypogastrium. Standard blood tests (including blood cell counts and total IgE) were within normal values. Ultrasonography displayed subcutaneous ovoid echogenic formation (14 × 5 × 5 mm) which was initially evaluated as hyperplastic lymph node or fibrolipoma and extirpated.

**Fig. 1:**
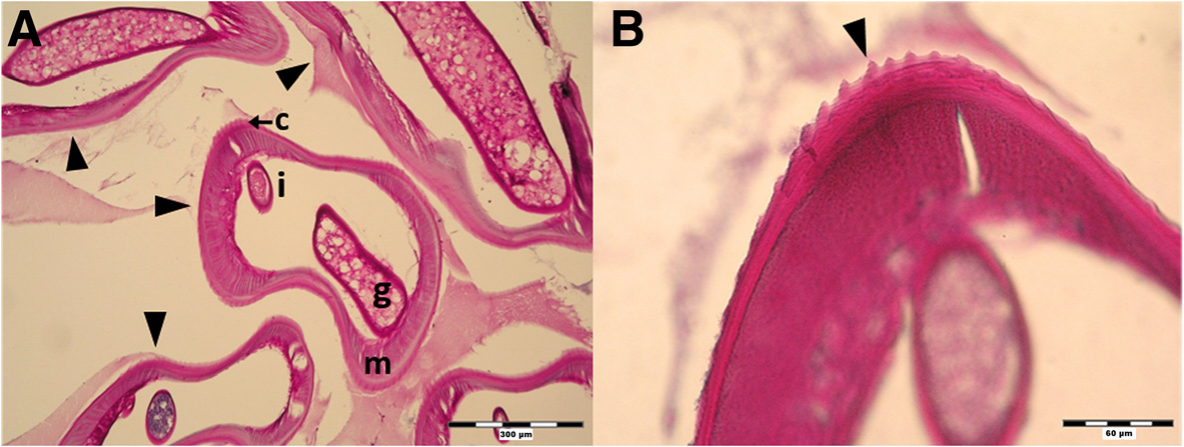
Histological section of subcutaneous formation extracted from hypogastrium (PAS staining; Case 1). **a** Section of the nodule containing the coiled worm (*arrowheads*) with visible genital tube (**g**), intestine (**i**), coelomyar musculature (**m**) and striated cuticle (**c**); 100 x. **b** Detailed view on longitudinal and transversal striation of the cuticle (*arrowhead*); 400 x

Histological examination of the extracted tissue revealed layered structure with massive peripheral accumulation of inflammatory (mostly eosinophil) cells with surrounded central space with a coiled roundworm sized 600–1000 μm in diameter. The worm cuticle showed transversal striation and longitudinal ridges arranged evenly with wide inter-ridge spaces exceeding their height. The worm musculature was that of coelomyarian type; the intestine and reproduction tube had parenchymatous tissue in its lumen (Fig. 1). Based on morphological features including the distribution of longitudinal ridges, the worm was determined as a larval/subadult worm of *Dirofilaria,* most likely of *D. repens*. Using DNA analysis, the *D. repens* was confirmed. Increased anti-filaria IgG antibodies levels were detected in blood samples obtained 1 and 2 months post extirpation; neither eosinophilia nor increased total IgE levels pointing to ongoing helminthiasis were measured.

### Case 2 (August 2014)

A homeless patient of Czech origin underwent surgery for abdominal hernia. There was a positive history of repeated mosquito bites but a negative history of travelling abroad. During the surgical intervention, a motile living worm appeared in the peritoneum.

The worm was extracted in toto, fixed in 4 % formaldehyde, washed and stored in 70 % ethanol.

**Fig. 2:**
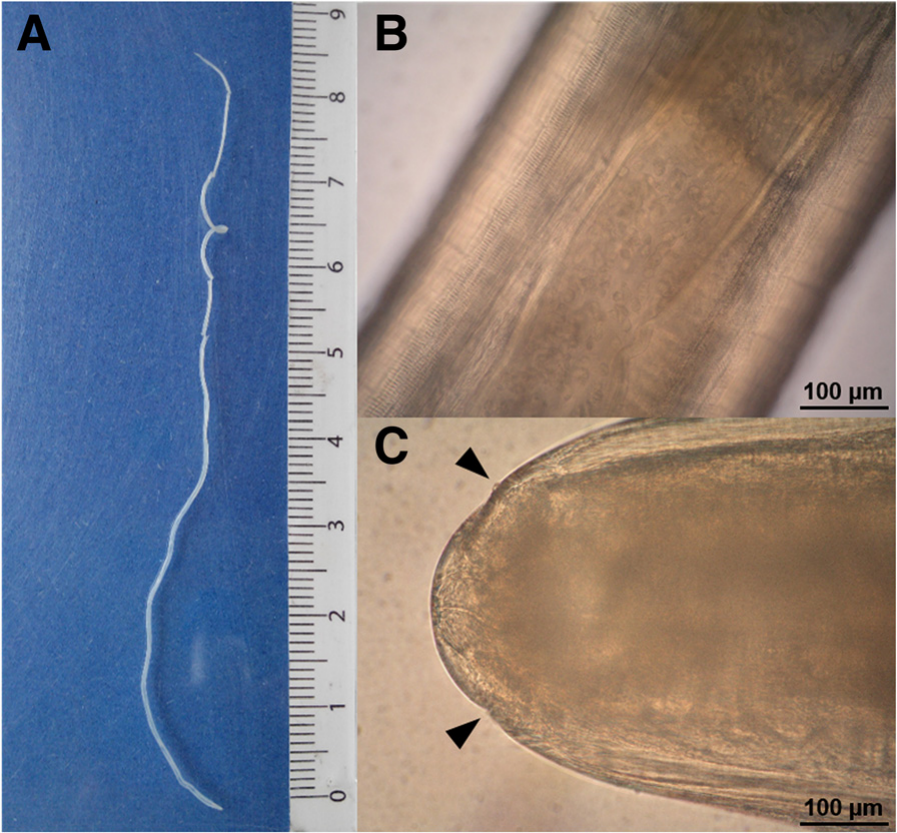
Subadult female of *Dirofilaria repens* found in the peritoneum of the patient during hernia surgery (Case 2). **a** General view of intact worm*.* **b** Detail on the cuticle with transversal and longitudinal striation, with visible uterus containing germinative cells; 200 x. **c** Anterior end of the worm with sensoric papillae (*arrowheads*); 200 x

A roundworm almost 10 cm long and up to 1 mm wide, with transversal cuticular striation and longitudinal ridges was identified. An underdeveloped female genital tract containing germinative cells within the uterus was observed (Fig. 2). Based on the morphology, *D. repens* was suspected and subsequently confirmed by DNA analysis. Anti-filaria IgG antibodies at a borderline level were detected in the serum taken two weeks after the surgery. Neither eosinophilia nor increased total IgE levels were measured.

### Case 3 (July 2013)

A patient of Czech origin manifested with a skin lesion of 15 mm in diameter above his left ankle, initially classified as a furuncle. The patient frequently travelled abroad, particularly to Germany, Austria, Poland and Slovakia 1 year prior to the disease presentation and to Kenya, Tanzania and Portugal more than 1 year earlier. The lesion developed at a site of an insect bite 3 weeks before the clinical examination. The lesion was surgically removed and two roundworms were extracted and fixed in 4 % formaldehyde.

The worms were 23 and 30 mm long and 1 mm wide, both with prominent longitudinal cuticular ridges on the entire body crossed with fine transversal striations. Simple body was narrowed with rounded anterior and posterior end. Based on the morphology the worms were classified as *Dirofilaria* spp. (suspected *D. repens*). DNA analysis was not performed. Borderline level of anti-filaria antibodies was detected in the serum sample taken 4 days after the surgery.

### Case 4 (December 2011)

A patient of Czech origin, without any travel history abroad, suffered from intermittent erythema and edema of proximal middle finger phalanx on the left hand and induration with erythema temporarily progressing up to hand dorsum for up to 3 weeks. Palpable mass on radial part of phalanx was found and incision followed. A filiform object approx. 4 cm long and at maximum 0.75 mm wide was extracted and fixed in 96 % ethanol.

**Fig. 3:**
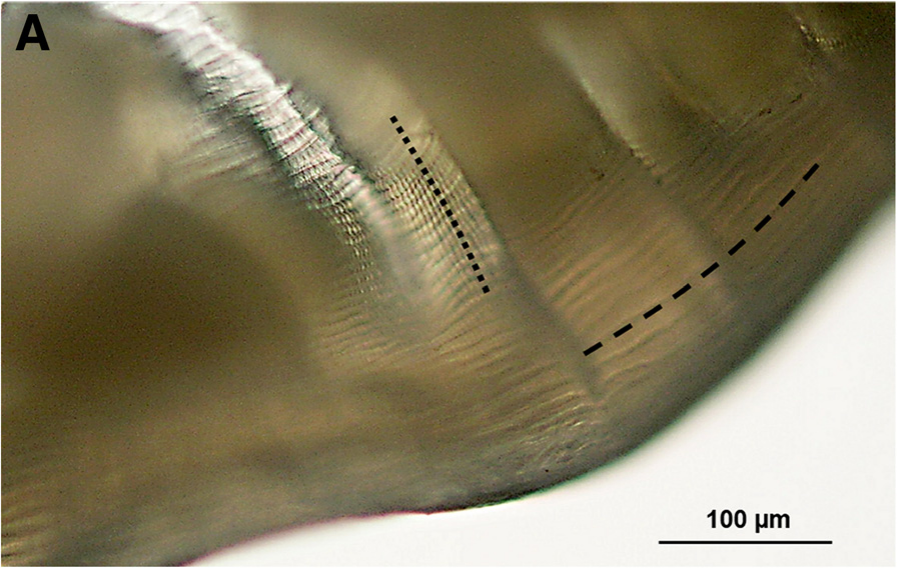
The worm extracted from the nodule on the left middle-finger of the patient (Case 4). **a.** Detail on cuticular structure with *Dirofilaria*-specific longitudinal ridges (**-----**) and transversal striation (**∙∙∙∙∙**); Photo P. Kotíková

A roundworm with longitudinal (sporadically branching) ridges and finer transversal stripes on the cuticle of entire body except anterior end was identified. Based on the morphology, the object was determined as *Dirofilaria* spp. (suspected *D. repens*; Fig. 3). The DNA analysis confirmed *D. repens*. Serological examination of the serum sample taken 1 day after intervention did not reveal anti-filaria IgG antibodies.

### Case 5 (June 2010)

A patient of Czech origin reporting frequent visits of Hungary, Slovakia and Croatia, and insect bites underwent surgical extraction of palpable lymph node (no details on symptoms and lymph node localization were provided, however trichinellosis or filariasis was suspected).

**Fig. 4:**
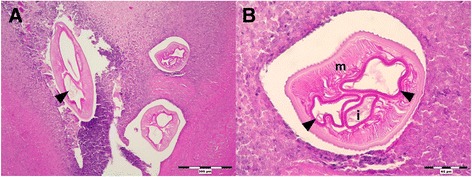
Histological sections of infected lymph node (PAS staining; Case 5). **a** Cross section of the nodule with parasite surrounded by inflammatory infiltration; uterus branches with developing microfilariae visible (*arrowhead*); 100 x. **b** Detail on longitudinal cuticle ridges; well-developed musculature (*m*); intestine (*i*).and uterus branches (*arrowheads*); 400 x

Histological examination of the lymph node showed several, mostly transverse, sections through a nematode of 125–500 μm in diameter, surrounded by inflammatory infiltration (Fig. 4a). The worm cuticle showed longitudinal ridges distributed over entire body with irregular distances; musculature of coelomyarian type, intestine and uterine tubes with developing larvae (microfilariae) were noted (Fig. 4). Based on histological examination, worm was identified as gravid female of *Dirofilaria* spp. (suspected *D. repens*). DNA analysis was not performed. No anti-filaria antibodies were detected in serum taken 1 month after intervention, however borderline level of specific antibodies was detected 1 and 3 months later.

### Summary

In all cases, observed morphological characteristics of either isolated or sectioned worms corresponded to the description of the genus *Dirofilaria*. Subsequent DNA isolation for species determination was not performed in case 3 (lack of material) and failed in case 5 (DNA damage as a result of tissue processing). The PCR amplicons of appropriate size were obtained in case 1 (histological section) and cases 2 and 4 (isolated worms). In all three analyzed samples, sequences revealed 97–99 % similarity to sequences of *D. repens* available in GenBank®. Representative sets of obtained sequences were submitted to GenBank® (accession numbers are provided in Table 2). Except for case 4, increased levels of anti-filaria IgG antibodies (either borderline or positive) were detected in the sera of patients. No significant IgG reactivity with other parasite antigens (*Toxocara canis, Trichinella spiralis, Echinococcus granulosus, Taenia saginata, Fasciola hepatica*) was detected in any case.

**Table 2:** Summary of the results obtained using particular diagnostic tools

Case No	Microscopy	DNA analysis				Serology
		Gene	Sequence lenght (bp)	Database accesion No	Identity (Database accesion No)	
1	*Dirofilaria* spp. (Susp. *D.repens*)	cox 1	682	*[GenBank: KR998257]*	99 % *D. repens [GenBank:* KF692102]	Positive
2	*Dirofilaria* spp. (Susp. *D.repens*)	cox1	482	*[GenBank: KR998258]*	99 % *D. repens* [*GenBank:* KF692102]	Borderline
		5S (153 bp)	153	*[GenBank: KR998254]*	97 % *D. repens* [*GenBank:* KC429769]	
		5S (247 bp)	247	*[GenBank: KR998255]*	99 % *D. repens [GenBank:* AJ242966]	
3	*Dirofilaria* spp. (Susp. *D.repens*)	-	-	-	-	Borderline
4	*Dirofilaria* spp. (Susp. *D.repens*)	cox 1	664	*[GenBank: KR998259]*	99 % *D. repens* [*GenBank:* KF692102]	Negative
		5S (153 bp)	153	*[GenBank: KR998256]*	99 % *D. repens* [*GenBank:* AJ242967]	
5	*Dirofilaria* spp. (Susp. *D.repens*)	-	-	-	-	Borderline

Diagnostic data on the patients are summarized in Table 2.

**Fig. 5:**
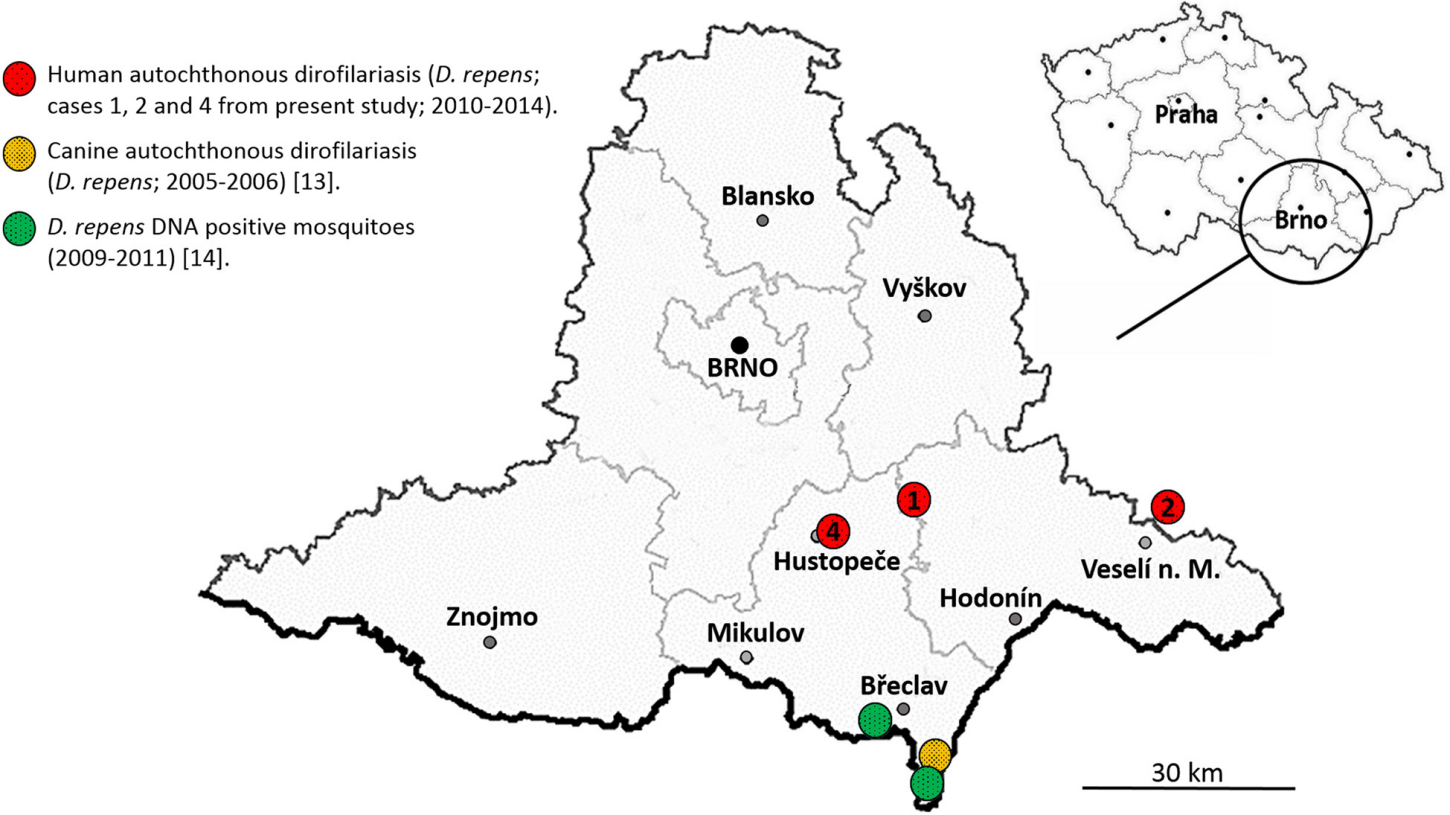
Geographical distribution of *Dirofilaria repens* records in the Czech Republic, South Moravian Region including findings from mosquitoes, dogs and humans

All the patients were of Czech origin. The patients without any history of foreign travel reside in the South Moravian Region of the CR, nearby the areas of *D. repens* infected dogs [13] and *D. repens* DNA positive mosquitoes (*Aedes vexans*) occurrence [14] (Fig. 5).

## Discussion

Human dirofilariosis is an important parasitic zoonosis, acquired from carnivores. Its recent emergence in Europe, for whatever reasons, is a prominent example of need for a One Health approach both in the diagnostics and control. Here, described cases of human dirofilariasis were diagnosed using three complementing diagnostic tools. After initial light microscopy investigation, DNA analysis and serological tests for specific antibodies were performed. Light microscopy is considered as reliable diagnostic tool for both, *Dirofilaria* genus and species determination: the roundworms are whitish and filiform; adult individuals are of variable length which depends on the sex and species (e.g. *D. repens* male 50–70 mm, female 100–170 mm). The parasites possess a simple terminal oral opening without lips or any sclerotized structures and an obtuse tail, in males equipped with two lateral allae and unequally sized spicules. The worm cuticle exhibits specific structure, including genus-specific transverse striation and species-specific longitudinal ridges [19, 20]. The distribution and appearance of longitudinal ridges represent reliable morphological markers to classify *Dirofilaria* species. In turn, both of the *Dirofilaria* species occurring in Europe could easily be distinguished by their different distribution of ridges. Cuticular ridges of *Nochtiella* subgenus worms including *D. repens* are arranged along the entire body while the cuticle of *D. immitis* worms (*Dirofilaria* subgenus) lacks these ridges except for ventral part of male hind tail [20]. The usual finding is that of single subadult worm but, occasionally, even mature worms can be found although human host is supposed to be unsuitable for their life cycle [21]. This is in line with our observation of developing microfilaria in female uterus in case 5, indicating a presence of a male worm even if it was not specifically detected. All the worms investigated in present study exhibited morphological signs of the *Dirofilaria* genus; the distribution of longitudinal cuticular ridges corresponded to the characteristics of *D. repens*.

In order to provide a definitive diagnosis on the molecular level, a range of PCR assays has been developed to detect the filarial DNA in definitive hosts, as well as in arthropod vectors, in the past decade. As a targets for amplification by nematode specific [18], filaria specific [16] or *Dirofilaria* specific [22, 23] primers by PCR (conventional or real-time), the regions of mitochodrial (*cox*1) [16–18, 22, 23] or nuclear genes (12S rDNA, 5SrDNA) [16, 18] are commonly used. In the present study, 5S rRNA gene and cox1 were used, combined with sequencing of PCR amplicons, either directly or after cloning [17, 18]. The DNA analysis was not performed in two cases (case 3 and 5) due to the lack of material and DNA damage, respectively. In all three samples processed for DNA analysis (cases 1, 2 and 4), revealed sequences that unequivocally confirmed the identity of *D. repens*.

Serology is an alternative method of diagnosing filariases (including dirofilariases) infection. For specific anti-filaria antibodies, detection using the antigens of *Acanthocheilonema* (syn. *Dipetalonema*) *vitae* are widely used [24]. In our study, both in-house prepared and commercially available *A. vitae* antigens were employed. Anti-filaria antibodies were detected in the sera of four patients, with a high level in single case with a subcutaneous location of worm, and borderline levels in three cases with worms found in the skin, peritoneum and lymph node. In one patient with finger soft tissues, infested anti-filaria antibodies were absent. The sera were supplied and tested in various periods after dirofilariasis was suspected or diagnosed (1 day to 4 months in particular cases). Thus, the time-frame of specific antibodies production could hardly be stated. Nevertheless, at least in one case, the repeated serological tests revealed delayed seroconversion 2 months after parasite removal. As in the case without anti-filaria antibodies, only serum taken 1 day post- parasite removal was tested, we suppose that potential delayed antibody production might appear, but escaped unnoticed.

Cases reported in our study represent the first records of human dirofilariasis in the CR. Since three of our patients had negative history of travel abroad, we propose that autochtonous human dirofilariosis has emerged in our country.

Recent emergence of autochthonous dirofilariasis was confirmed in several areas in Europe, where the disease was previously considered to be imported from endemic countries [6–11]. The occurrence of dirofilariasis depends on the availability of susceptible vector species and the climate suitable for successful intra-mosquito development. It is supposed that the emergence of dirofilariasis in previously non-endemic areas reflects the vector(s) expansion and/or increased parasite survival due to the ongoing climate changes as well as increased global movement (travelling, trade and transport in particular) [1]. All three patients with autochthonous dirofilariasis resided the South Moravian Region, sharing a border with Slovakia and Austria, where human dirofilariasis was recorded. The region is characterized by high annual mean temperatures and high mosquito abundance, with *A. vexans* present annually as eudominant species [25]. Although absent in most of the territory of the CR, *D. repens* is locally well established in domestic dog population along Morava and Dyje (Thaya) rivers [12, 15], representing the northeast expansion of the parasite from Pannonian lowlands of Slovakia [26] and Austria [27]; infected populations of *Aedes vexans* mosquitoes have been recently detected in the same area [14].

## Conclusions

This is the first report of autochtonous human *Dirofilaria* infections in the CR. Based on our observations, we confirm the natural setting of *D. repens* in the area where it may represent a significant risk factor for public health. We propose that dirofilariasis is endemic in the Southeast part of the country - Southern Moravia and should be considered in the differential diagnosis of subcutaneous nodules (or rarely other organ nodular affections) not only in patients who travelled abroad but also in those without any travel history.

## Availability of data and materials

Patient samples (sera, paraffin embedded tissue blocks and extracted worms) and complete data obtained by diagnostic methods applied are deposited in National Reference Laboratory for Tissue Helminthoses, General University Hospital in Prague (Prague, Czech Republic). Corresponding microphotographs of extracted tissues/worms are included in MS (Figs. 1, 2, 3, 4). Representative sets of obtained nucleic acid sequences were submitted to GenBank® (NCBI) and accession numbers are included in MS (Table 2).

## Consent

The study is retrospective. Only archived patients’ data and samples processed for diagnostic purposes were used. Prior the processing, written consent with examination and diagnostic tests, as well as with potential further use of the data and samples for scientific or educational purposes including publication of anonymized data was provided by each particular patient.

All the patients’ data and material used in the study are entirely unidentifiable. There are no details on individuals reported within the manuscript thus the need of any additional consent for data publication was waived by the Ethics committee of the General University Hospital in Prague, Czech Republic that has approved the study.
